# Chemical Components and Antioxidant Activity of *Geotrigona* sp. and *Tetragonisca fiebrigi* Stingless Bee Cerumen Reduce Juglone-Induced Oxidative Stress in *Caenorhabditis elegans*

**DOI:** 10.3390/antiox12061276

**Published:** 2023-06-15

**Authors:** Isamara Carvalho Ferreira, Raíssa Cristina Darroz Côrrea, Sarah Lam Orué, Daniel Ferreira Leite, Paola dos Santos da Rocha, Claudia Andrea Lima Cardoso, Rosilda Mara Mussury, Patricia Vit, Kely de Picoli Souza, Edson Lucas dos Santos, Jaqueline Ferreira Campos

**Affiliations:** 1 Research Group on Biotechnology and Bioprospecting Applied to Metabolism (GEBBAM), Federal University of Grande Dourados, Rodovia Dourados-Itahum, Km 12, Dourados 79804-970, MS, Brazil; isamara.ferreira137@academico.ufgd.edu.br (I.C.F.); raissa.correa416@academico.ufgd.edu.br (R.C.D.C.); sarah.orue005@academico.ufgd.edu.br (S.L.O.); daniel.leite059@academico.ufgd.edu.br (D.F.L.); paolasantosrocha@ufgd.edu.br (P.d.S.d.R.); kelypicoli@ufgd.edu.br (K.d.P.S.); edsonsantos@ufgd.edu.br (E.L.d.S.); 2 Course of Chemistry, State University of Mato Grosso do Sul, Rodovia Dourados-Itahum, Km 12, Dourados 79804-970, MS, Brazil; claudia@uems.br; 3 Faculty of Biological and Environmental Sciences, Federal University of Grande Dourados, Rodovia Dourados-Itahum, Km 12, Dourados 79804-970, MS, Brazil; maramussury@ufgd.edu.br; 4 Apitherapy and Bioactivity, Food Science Department, Faculty of Pharmacy and Bioanalysis, Universidad de Los Andes, Mérida 5101, Venezuela

**Keywords:** bioprospecting, meliponines, oxidative stress

## Abstract

Cerumen is a bee product produced exclusively by stingless bees, resulting from a mixture of beeswax and plant resins. The antioxidant activity of bee products has been investigated since oxidative stress is associated with the onset and progression of several diseases that can lead to death. In this context, this study aimed to investigate the chemical composition and antioxidant activity of cerumen produced by the *Geotrigona* sp. and *Tetragonisca fiebrigi* stingless bees, in vitro and in vivo. The chemical characterization of cerumen extracts was performed by HPLC, GC, and ICP OES analyses. The in vitro antioxidant potential was evaluated by DPPH^•^ and ABTS^•+^ free radical scavenging methods, and in human erythrocytes subjected to oxidative stress with AAPH. In vivo, the antioxidant potential was evaluated in *Caenorhabditis elegans* nematodes subjected to oxidative stress with juglone. Both cerumen extracts presented phenolic compounds, fatty acids, and metallic minerals in their chemical constitution. The cerumen extracts showed antioxidant activity by capturing free radicals, reducing lipid peroxidation in human erythrocytes, and reducing oxidative stress in *C. elegans*, observed by the increase in viability. The results obtained indicate that cerumen extracts from *Geotrigona* sp. and *Tetragonisca fiebrigi* stingless bees may be promising against oxidative stress and associated diseases.

## 1. Introduction

Stingless bees are included in the subfamily Meliponinae (Hymenoptera, Apidae) consisting of 58 genera and more than 550 species [1]. These meliponines play a crucial role in plant pollination, being responsible for about 40–90% of the pollination of native species or those cultivated in the tropics [2]. Furthermore, bee products from stingless bees, which include honey, propolis, wax, and cerumen, are used in folk medicine [3] and have pharmacological activities reported in the literature, such as antioxidant action [4,5,6,7].

The antioxidant activity of bee products can be related to the presence of bioactive molecules [8], including phenolic compounds, aromatic acids, alcohols, and sugars [9], that are capable of neutralizing oxidative stress effects [10]. Oxidative stress results from the imbalance between the production of reactive species, such as oxygen, and the number of antioxidant agents to neutralize them [11]. This state of imbalance can be associated with the onset and/or progression of several diseases, such as diabetes, cardiovascular diseases, and cancer, which are among the leading causes of death in the world today [12,13].

From this perspective, we highlight the stingless bees *Geotrigona* sp. (Moure, 1943) and *Tetragonisca fiebrigi* (Schwarz, 1938), distributed in different countries of Latin America, as producers of cerumen [14,15]. Cerumen is a product elaborated exclusively by meliponines, consisting of a mixture of beeswax and plant resins [16]. Previous studies have described the biological activities of cerumen from other bee species, such as antioxidant activity [4]. However, no studies report the chemical composition and the biological potential of the cerumen produced by *Geotrigona* sp. and *Tetragonisca fiebrigi.*

To evaluate the pharmacological effects of different substances, especially on the modulation of oxidative stress, the *Caenorhabditis elegans* model has been widely used due to the homology of its genes with human genes [17]. In this context, this study aimed to investigate the chemical composition and evaluate the antioxidant activity of the cerumen produced by the stingless bees *Geotrigona* sp. and *T. fiebrigi* in vitro and nematodes *C. elegans*.

## 2. Materials and Methods

### 2.1. Preparation of Ethanolic Extracts of Cerumen from Geotrigona sp. (EEC-G) and Tetragonisca fiebrigi (EEC-T)

The cerumen of the stingless bee *Geotrigona* sp. was purchased in Quimís, Manabí province, Ecuador, and that of *Tetragonisca fiebrigi* was collected in Dourados, Mato Grosso do Sul, Brazil, after Dalmo Henrique Franco Silva specialist identification. The cerumen was stored at −20 °C until the preparation of the extract.

The cerumen samples were solubilized at a ratio of 4.5 mL with 80% ethanol for each gram of cerumen [18]. The solutions were kept in a water bath at 70 °C in a closed container and periodically homogenized until complete dissolution. Finally, the material was filtered to obtain ethanolic extracts of cerumen from *Geotrigona* sp. (EEC-G) and *T. fiebrigi* (EEC-T).

### 2.2. Chemical Composition

#### 2.2.1. HPLC Analysis

The phenolic compounds were identified and quantified using ultra-high-efficiency liquid chromatography (UHPLC) coupled to a diode array detector (DAD) with wavelength monitoring between 200 and 800 nm. A Shimadzu Shim-pack XR-ODS column (Shimadzu, Kyoto, Japan) with a particle size of 2.2 µm and a particle size of 2 mm × 75 mm was used. The injection rate employed was 2 µL with a 0.4 mL/min flow rate. The gradient used consisted of (A) water with 0.1% acetic acid (*v/v*) and (B) acetonitrile with 0.1% acetic acid (*v/v*). Identification was performed using analytical standards (3,4-hydroxycinnamic acid, catechin, gallic acid, ferulic acid, coumaric acid, maleic acid, rutin, quercertin, apigenin, and vanillin) obtained from Merck (Darmstadt, Germany) A curve was developed with the analytical standards at concentrations between 1 and 1000 µg/mL for quantification.

#### 2.2.2. GC Analysis

The fatty acid content was determined using the method described by Bligh and Dier [19]. For fatty acid extraction, 1 mL of EEC-G or EEC-T was mixed with 10 mL of chloroform, 20 mL of methanol, and 8 mL of distilled water (1:2:0.8) and kept in constant homogenization for 30 min. Subsequently, 10 mL of chloroform and 10 mL of the 1.5% sodium sulfate solution were added and kept in homogenization for two minutes. Then, the excess water and methanol were removed from the tubes.

The material remained in an oven at 100 °C until the evaporation of the solvent. After drying, the material was solubilized, and the sample was methylated with 0.25 mol/L sodium methylate in diethyl ether methanol (1:1). Gas chromatography (GC) equipped with a flame ionization detector was performed for the analyses. An SP2560 30 m × 0.25 mm × 0.20 m column was used with a temperature gradient: 80 °C, 0 min, 7 °C/min up to 240 °C; injector (1/30 split) at 220 °C and detector at 250 °C. Hydrogen was used as the carrier gas (2.0 mL/min) with an injection volume of 1 µL. Peak identification was performed by comparison with FAME C8–C22 methylated fatty acid standards. Quantification was performed by external standardization.

#### 2.2.3. ICP-OES Analysis

The metal mineral content was determined by acid extraction using nitric acid (HNO_3_) and hydrogen peroxide (H_2_O_2_) 30%. To determine the metal content, the samples were prepared with deionized water. The glassware and bottles used for storage and/or sample preparation were decontaminated with nitric acid (HNO_3_) 10% (*v/v*) beforehand, incubated for 24 h, and then washed with deionized water. For the assay, EEC-G or EEC-T were subjected to acid extraction, performed in borosilicate glass tubes in a digester block with a timer (SL-25/40, Solab, Piracicaba, Brazil), and subjected to pre-digestion with 3.5 mL of HNO_3_ for 24 h at room temperature (overnight). After this period, they were digested for 45 min at 150 °C. Then, 1.5 mL H_2_O_2_ was added and kept for another 45 min in the digester block under the same heating. The blank only contained the mixture of HNO_3_ and H_2_O_2_.

The obtained solution was diluted to 10 mL with deionized water and kept at rest for cooling. The analyses were performed by inductively coupled plasma optical emission spectrometry (ICP-OES). The equipment was adjusted using solutions between 10 and 100 µg/L containing the elements Al, Ba, Ca, Cu, Fe, K, Mg, Mn, Na, P, S, and Zn and the following operational parameters: radiofrequency power 1500 W, radiofrequency generator 27.12 MHz, sample aspiration flow rate 1.5 mL/min, argon flow rate (L/min) 15.00 (plasma), and 1.00 (auxiliary) 0.45 (nebulizer). The multi-element determination of the sample was expressed in mg/L and the analyses were performed in triplicate.

### 2.3. In Vitro Antioxidant Activity

#### 2.3.1. 2,2-Diphenyl-1-picrylhydrazyl (DPPH^•^) Free Radical Scavenging

For the in vitro evaluation of the antioxidant activity of EEC-G and EEC-T, the DPPH- free radical capture assay was performed [20]. For this, 200 µL of EEC-G or ECC-T (0.001–3 mg/mL) was added to 1800 µL of 0.11 mM DPPH^•^. The solution was kept in the dark at room temperature for 30 min. Then, the absorbance (Abs) was measured in a spectrophotometer at 517 nm. Ascorbic acid and butylated hydroxytoluene (BHT) were used as the standard. Three independent experiments were performed in duplicate. The percentage of DPPH^•^ capture was calculated according to the following equation:

DPPH-capturing activity (%) = (1 − Abs sample/Abs control) × 100,(1)

and the concentration able to inhibit 50% of the free radical (IC_50_) was calculated.

#### 2.3.2. Elimination of the Free Radical 2,2′-Azino-bis(3-ethylbenzothiazoline-6-sulfonic acid) (ABTS^•+^)

The antioxidant activity of the cerumen extracts was also evaluated using a 2,2′-azinobis(3-ethylbenzothiazoline-6-sulfonic acid) radical capture assay (ABTS^•+^) [21]. The ABTS^•+^ radical was prepared by adding 88 µL of a potassium persulfate solution (140 mM) to 5 mL of the aqueous ABTS solution (7 mM). After 12 h, this solution was diluted in absolute ethanol until an absorbance of 0.700 ± 0.05 at 734 nm was obtained. Then, 20 µL of EEC-G or EEC-T (0.001–2 mg/mL) was added to 1980 µL of ABTS^•+^ radical solution and incubated for 6 min in the dark at room temperature. The absorbance was measured in a spectrophotometer at 734 nm.

Ascorbic acid and BHT were used as reference antioxidants. Three independent experiments were performed in duplicate. The percentage of ABTS^•+^ radical inhibition was calculated according to the following equation:

ABTS radical inhibition (%) = ((Abs control − Abs sample)/Abs control) × 100,(2)

and the concentration able to inhibit 50% of the free radical (IC_50_) was calculated.

### 2.4. Antioxidant Activity in Human Erythrocytes

After approval by the Research Ethics Committee of the University Center of Grande Dourados (UNIGRAN), Brazil (CEP process number 123/12-UNIGRAN), 20 mL of peripheral blood was collected from a healthy donor in tubes containing sodium citrate. The material was centrifuged at 1500 rpm for 10 min. Subsequently, the blood plasma and leukocyte layers were discarded. The erythrocytes were washed three times with 0.9% sodium chloride (NaCl) solution. A 10% red cell suspension in 0.9% NaCl was prepared for the assays.

#### 2.4.1. Hemolysis and Oxidative Hemolysis

The anti-hemolytic assay was performed on human erythrocytes to evaluate the ability of cerumen extracts to provide antioxidant protection in human cells [5]. For this, a 10% erythrocyte suspension was pre-incubated at 37 °C for 30 min in the presence of different concentrations of EEC-G or EEC-T (0.01–1 mg/mL). Then, to evaluate the effect of the extracts on hemolysis and oxidative hemolysis, 0.9% NaCl or the oxidizing agent 2,2′-Azobis(2-amidinopropane) dihydrochloride (AAPH) 50 mM, respectively, was added. The samples were kept at 37 °C for 2 h under constant stirring. After this period, the samples were centrifuged at 3000 rpm for 5 min, and 200 µL of the supernatant was added to 1800 µL of 0.9% NaCl. The absorbance was measured in a spectrophotometer at 540 nm. A 0.36% ethanol solution was used as a solvent control. A solution of ascorbic acid (0.01–1 mg/mL) was used as a positive control. Total erythrocyte hemolysis was induced with distilled water. Three independent experiments were performed in duplicate. The percentage of hemolysis was determined by the equation:

Hemolysis (%) = (Abs sample/Abs total hemolysis) × 100.(3)

#### 2.4.2. Malondialdehyde Dosage (MDA)

To evaluate the efficiency of cerumen extracts on lipid peroxidation, the MDA dosage was realized in human erythrocytes subjected to oxidative stress induced with AAPH [5]. For this, a 10% erythrocyte suspension was pre-incubated at 37 °C for 30 min with different concentrations of EEC-G or EEC-T (0.01–1 mg/mL). Subsequently, a 50 mM AAPH solution was added and the samples were kept at 37 °C for 2 h under constant stirring. After this period, the samples were centrifuged at 3000 rpm for 5 min. Then, 500 µL of the supernatant was collected and added to 1 mL of 10 nM thiobarbituric acid (TBA). All samples were incubated at 96 °C for 45 min and then cooled for 15 min. After this period, 4 mL of n-butyl alcohol was added to the samples, which were centrifuged at 3000 rpm for 5 min. The absorbance of 2 mL of the supernatant was measured in a spectrophotometer at 532 nm. A 0.36% ethanol solution was used as a solvent control. Ascorbic acid (0.01–1 mg/mL) was used as a positive control. MDA at 20 µM was used as a standard. Three independent experiments were performed in duplicate. MDA content was expressed in nmol/mL, obtained by the following equation:

MDA (nmol/mL) = Abs sample × (20 × 220.32/Abs MDA standard).(4)

### 2.5. Antioxidant Activity in C. elegans

To perform the in vivo assays, the wild-type strain N2 of *C. elegans* nematodes, obtained from the *Caenorhabditis* Genetics Center (University of Minnesota, Minneapolis, MN, USA), was used. The animals were kept in Petri dishes containing Nematode Growth Medium (NGM) agar and fed with *Escherichia coli* OP50 bacteria. For the assays, the nematodes were synchronized by treatment with hypochlorite (1%) and sodium hydroxide (NaOH 5 M) [22].

#### 2.5.1. Acute Toxicity Assay

The acute toxicity assay was performed to evaluate the effect of EEC-G and EEC-T on the viability of *C. elegans* [23]. For this, 10 to 20 synchronized nematodes at the L4 stage were transferred to 96-well plates containing M9 medium. Then, different concentrations of EEC-G or EEC-T (0.005–1 mg/mL) were added. The treated nematodes were kept in BOD at 20 °C for 24 h. After this period, the viability of the nematodes was evaluated under a stereomicroscope. The individuals were considered alive when they moved after being touched with a platinum micro spatula. Three experiments were performed in triplicate.

#### 2.5.2. Oxidative Stress Resistance Assay

To evaluate the effect of EEC-G and EEC-T on the oxidative stress induced by 5-hydroxynaphthalene-1,4-dione (juglone) in *C. elegans*, the oxidative stress resistance assay was performed [24]. For this, 10 to 20 nematodes at the L4 stage were transfected into 96-well plates containing liquid M9 medium. The nematodes were pretreated with different concentrations of EEC-G or EEC-T (0.01–1 mg/mL) for 1 h at 20 °C. Then, the animals were exposed to the oxidizing agent juglone (40 µM) and incubated at 20 °C for 24 h. After this period, the nematode viability was assessed using a stereomicroscope. The individuals were considered alive when they moved after being touched with a platinum micro spatula. Three experiments were performed in triplicate.

### 2.6. Statistical Analysis

The data obtained were expressed as the mean ± standard error of the mean (SEM). The 50% inhibitory concentration (IC_50_) with 95% confidence limits was determined by non-linear regression. For analysis and comparison between the experimental groups, univariate analysis of variance (ANOVA) was used, followed by Dunnett’s post hoc test. The results were considered significant when *p* ≤ 0.05.

## 3. Results

### 3.1. Chemical Composition

The phenolic compounds, fatty acids, and metal minerals identified in EEC-G and EEC-T are presented in Table 1. HPLC analysis revealed the presence of different phenolic compounds for EEC-G and EEC-T. Among the phenolic compounds present in the cerumen extracts, catechin for EEC-G and rutin for EEC-T were the most noteworthy.

The GC analysis showed no difference in the composition of fatty acids between the cerumen extracts. EEC-G and EEC-T presented 14 fatty acids, with an emphasis on palmitic acid. The ICP OES analysis did not reveal any difference in the composition of metallic minerals between the cerumen extracts. EEC-G and EEC-T showed 12 metallic minerals, including copper, magnesium, and zinc.

### 3.2. In Vitro Antioxidant Activity

EEC-G and EEC-T showed antioxidant activity by scavenging DPPH^•^ and ABTS^•+^ free radicals (Table 2).

In DPPH^•^ free radical scavenging assay, EEC-G showed lower IC_50_ when compared to EEC-T. In the ABTS^•+^ free radical scavenging assay, EEC-T showed two times lower IC_50_ than EEC-G.

### 3.3. Antioxidant Activity in Human Erythrocytes

**Figure 1:**
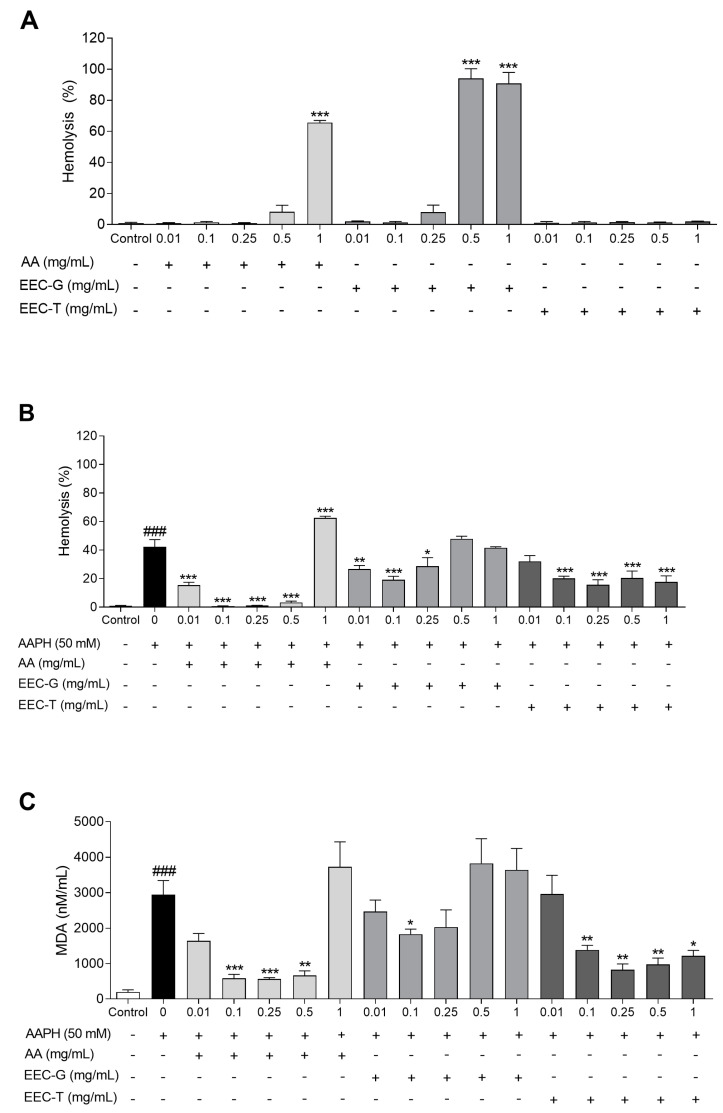
Antioxidant activity in human erythrocytes: (**A**) percentage of hemolysis of human erythrocytes treated with different concentrations of ascorbic acid, EEC-G, and EEC-T (0.01–1 mg/mL); (**B**) percentage of hemolysis AAPH and (**C**) MDA content of human erythrocytes treated with different concentrations of ascorbic acid, EEC-G, and EEC-T (0.01–1 mg/mL) and induced to oxidative stress with the oxidant agent AAPH. AA: ascorbic acid; EEC-G: ethanolic extract of *Geotrigona* sp. cerumen; EEC-T: ethanolic extract of *Tetragonisca fiebrigi* cerumen; AAPH: 2,2′-Azobis(2-amidinopropane) dihydrochloride; MDA: malondialdehyde. +: presence; −: absence. # versus control; * versus 0 (AAPH 50 mM); ^###^ *p* < 0.001; * *p* < 0.05; ** *p* < 0.01; *** *p* < 0.001. Values are expressed as the mean ± SEM. Three independent experiments were performed in duplicate.

EEC-G and EEC-T showed antioxidant activity in human erythrocytes (Figure 1). EEC-T did not promote hemolysis in human erythrocytes at the evaluated concentrations when compared to erythrocytes incubated with 0.9% NaCl only (control). However, EEC-G induced hemolysis in human erythrocytes at the concentrations of 0.5 and 1 mg/mL in a similar manner to ascorbic acid at the concentration of 1 mg/mL (Figure 1A).

When human erythrocytes were incubated with the oxidizing agent AAPH, EEC-T reduced hemolysis at concentrations of 0.1, 0.25, 0.5, and 1 mg/mL by 52.37%, 62.92%, 51.58%, and 58.20%, respectively, when compared to erythrocytes incubated with AAPH alone. EEC-G reduced hemolysis in human erythrocytes subjected to oxidative stress with the oxidizing agent AAPH at concentrations of 0.01, 0.1, and 0.25 mg/mL by 36.78%, 54.80%, and 32.02%, respectively, compared to erythrocytes incubated with AAPH only (Figure 1B).

EEC-T reduced the lipid peroxidation of human erythrocytes promoted by oxidative stress induced by the oxidizing agent AAPH, observed by lower MDA content generated at concentrations of 0.1, 0.25, 0.5, and 1 mg/mL by 52.81%, 72.01%, 66.66%, and 58.48%, respectively, compared to erythrocytes incubated with AAPH only. EEC-G reduced MDA content at the concentration of 0.1 mg/mL by 37.86% compared to erythrocytes incubated with AAPH alone (Figure 1C).

### 3.4. Acute Toxicity and Antioxidant Activity in C. elegans

**Figure 2:**
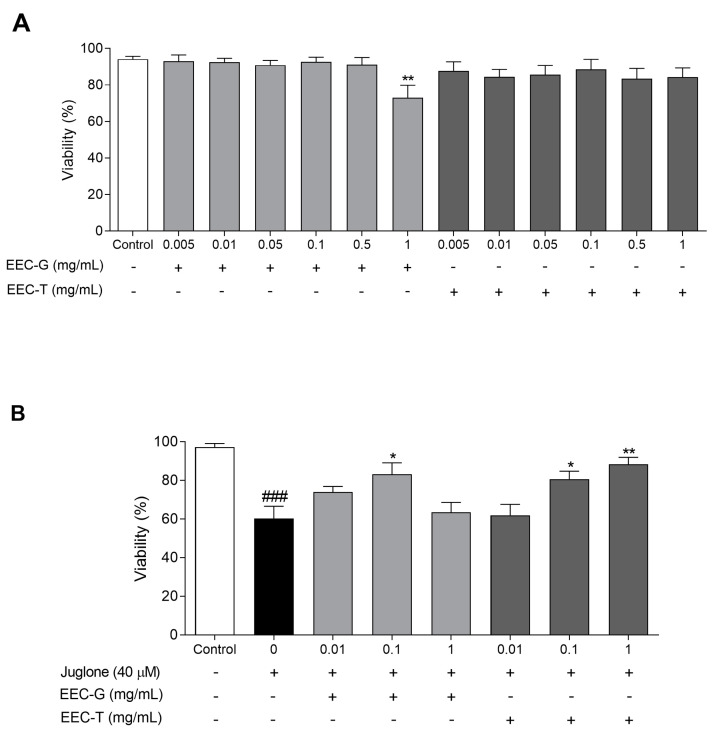
Acute toxicity and antioxidant activity in *C. elegans*: (**A**) percentage of viability of *C. elegans* treated with different concentrations of EEC-G and EEC-T (0.005–1 mg/mL); (**B**) percentage of viability of *C. elegans* treated with different concentrations of EEC-G and EEC-T (0.01–1 mg/mL) and induced oxidative stress with the oxidant agent juglone. EEC-G: ethanolic extract of *Geotrigona* sp. cerumen; EEC-T: ethanolic extract of *Tetragonisca fiebrigi* cerumen. +: presence; −: absence. # versus control; * versus 0 (juglone 40 µM); ^###^ *p* < 0.001; * *p* < 0.05; ** *p* < 0.01. Values are expressed as the mean ± SEM. Three independent experiments were performed in triplicate.

The effect of EEC-G and EEC-T on *C. elegans* viability is shown in Figure 2A. EEC-T showed no toxicity at the evaluated concentrations. However, EEC-G reduced the viability of *C. elegans* at the concentration of 1 mg/mL by 22.43%.

EEC-G and EEC-T showed antioxidant activity in *C. elegans* exposed to the oxidizing agent juglone (Figure 2B). EEC-T reduced the oxidative stress damage promoted by juglone in *C. elegans*, observed by the increase in viability at concentrations of 0.1 and 1 mg/mL by 33.87% and 46.63%, respectively, when compared to nematodes treated with juglone only. EEC-G increased viability at 0.1 mg/mL by 38.04% compared to nematodes treated with juglone only.

## 4. Discussion

This study shows, for the first time, the chemical composition and the antioxidant activity of the cerumen of stingless bees *Geotrigona* sp. and *T. fiebrigi* in vitro, on human erythrocytes, and in vivo, on *C. elegans* nematodes.

The chemical constitution of *Geotrigona* sp. and *T. fiebrigi* showed phenolic compounds, fatty acids, and metallic minerals. The chemical profile of the cerumen differed mainly in their phenolic constituents. EEC-G presented a higher proportion of flavonoid catechin, while EEC-T presented a higher concentration of flavonoid rutin. Among the fatty acids, palmitic acid stood out, and among the metallic minerals, manganese, zinc, copper, and iron were present in both extracts.

Phenolic compounds are described by their antioxidant potential, attributed to their chemical structure, consisting of an aromatic ring and free hydroxyls. Thus, these hydroxyls can donate their electrons to stabilize radical molecules [25].

The EEC-G showed better antioxidant activity by capturing the DPPH^•^ radical, while the EEC-T showed better antioxidant activity by capturing the ABTS^•+^ radical. The difference in the response of cerumen extracts is probably related to its phenolic profile, since the rutin present in EEC-T is a glycoside that has the flavonolic aglycone quercetin (lipophilic) and the disaccharide rutinose (hydrophilic) in its chemical structure, which together have many free hydroxyls and amphiphilic characteristics, which makes the antioxidant result more evident using the ABTS^•+^ free radical scavenging method [26,27].

Besides stabilizing radical molecules, phenolic compounds can also modulate the activity of antioxidant enzymes, contributing to the response to oxidative stress [25]. EEC-T reduced oxidative stress at concentrations of 0.1 to 1 mg/mL in human erythrocytes, observed by the lower percentage of hemolysis and corroborated by the lower malondialdehyde content generated. Rutin, only present in EEC-T, besides eliminating free radicals directly, is also able to increase the activity of the antioxidant enzyme glutathione peroxidase (GPx) by increasing the production of glutathione reductase (GR) responsible for maintaining the substrate for GPx action. Rutin also inhibits xanthine oxidase, which is involved in the generation of free radicals [28].

Additionally, antioxidant enzymes such as superoxide dismutase and catalase depend on metallic minerals such as iron, copper, zinc, and manganese for their function, which were observed in the chemical spectra of both extracts. Therefore, the inhibition of free radicals directly, together with the increase in the activity of antioxidant enzymes, may contribute to the reduction in lipid peroxidation observed in human erythrocytes submitted to oxidative stress.

EEC-G showed an antioxidant effect at a 0.1 mg/mL concentration and pro-oxidant effect at higher concentrations (0.5 and 1 mg/mL). Some antioxidants, such as ascorbic acid, have been described to have pro-oxidant potential at high concentrations [29]. Catechin, one of the EEC-G constituents, has been described as having a pro-oxidant action by its ability to induce pore formation that increases mitochondrial permeability [30]. Moreover, its pro-oxidant activity can be attributed to the increase in catechin-derived oxidants after the initial elimination of the superoxide radical anion [31,32].

In *C. elegans*, EEC-T ameliorated juglone-induced oxidative stress at concentrations of 0.1 and 1 mg/mL, and EEC-G only showed activity at 0.1 mg/mL and reduced viability at the highest concentration (1 mg/mL), probably due to its pro-oxidant characteristic at high concentrations.

Juglone is an organic compound capable of generating large amounts of the radical superoxide anion, which induces oxidative stress and reduces nematode viability [33]. During the redox imbalance promoted by the oxidizing agent juglone, signaling pathways, such as DAF-16/FOXO, may have been upregulated by the chemical compounds present in the cerumen extracts. DAF-16 activation increases the gene expression of *sod-3*, which triggers the activity of the mitochondrial antioxidant system through the conversion of the superoxide radical anion by the SOD-3 enzyme to hydrogen peroxide, which is considered less deleterious and, subsequently, can be converted into water and oxygen by catalase (CAT) and GPx enzymes [34].

In previous studies, it was shown that rutin, also present in EEC-T, reduced the levels of reactive oxygen species, activated DAF-16 migration, and increased *sod-3* expression in *C. elegans* [35,36,37]. Furthermore, in other biological models, rutin reduced MDA levels and increased the activity of antioxidant enzymes [38,39].

Additionally, it has been reported that palmitic acid and oleic acid, also present in *Geotrigona* sp. and *T. fiebrigi*, extracts, can increase the resistance to oxidative stress in *C. elegans* by activating DAF-16 [40,41]. Moreover, the presence of other constituents in the extracts, such as manganese, may contribute to the efficiency in the activity of the antioxidant enzyme SOD-3 in *C. elegans*.

## 5. Conclusions

Together, these results show, for the first time, the chemical composition and the effect of cerumen from *Geotrigona* sp. and *T. fiebrigi* stingless bees on the reduction of oxidative stress in human erythrocytes and *C. elegans*. Our results provide new perspectives for the development of future studies investigating the mechanisms of the antioxidant action of these products, as well as for potential use in diseases associated with oxidative stress.

## Figures and Tables

**Table 1:** Phenolic compounds, fatty acids, and metallic minerals were identified in the ethanolic extract of cerumen from *Geotrigona* sp. (EEC-G) and *Tetragonisca fiebrigi* (EEC-T).

Compounds	EEC-G (mg/L)	EEC-T (mg/L)
*Phenolic compounds*		
3,4-hydroxycinnamic acid	3.040 ± 0.032	-
Catechin	10.000 ± 0.044	-
Gallic acid	-	6.473 ± 0.020
Rutin	-	12.993 ± 0.022
Vanillin	4.020 ± 0.035	-
*Dicarboxylic acids*		
Maleic acid	1.970 ± 0.023	-
*Fatty acids*		
Caprylic acid	0.303 ± 0.003	0.347 ± 0.003
Capric acid	1.607 ± 0.007	1.503 ± 0.003
Lauric acid	2.590 ± 0.012	2.543 ± 0.009
Myristic acid	9.973 ± 0.035	10.583 ± 0.015
Pentadecanoic acid	0.103 ± 0.003	0.103 ± 0.003
Palmitic acid	37.193 ± 0.143	35.953 ± 0.046
Margaric acid	0.103 ± 0.003	0.107 ± 0.003
Stearic acid	10.983 ± 0.020	11.447 ± 0.009
Arachidic acid	3.347 ± 0.020	3.417 ± 0.018
Behenic acid	3.067 ± 0.023	2.987 ± 0.015
Myristoleic acid	7.447 ± 0.035	7.107 ± 0.020
Palmitoleic acid	8.683 ± 0.035	9.173 ± 0.032
Oleic acid	12.083 ± 0.071	12.027 ± 0.032
Linoleic acid	13.710 ± 0.059	13.523 ± 0.020
Linolenic acid	14.237 ± 0.041	14.023 ± 0.024
*Metallic minerals*		
Aluminum (Al)	0.713 ± 0.012	0.593 ± 0.009
Barium (Ba)	0.263 ± 0.003	0.293 ± 0.009
Calcium (Ca)	1.510 ± 0.017	1.343 ± 0.231
Copper (Cu)	0.260 ± 0.006	0.290 ± 0.017
Iron (Fe)	0.713 ± 0.012	0.620 ± 0.015
Potassium (K)	1.623 ± 0.024	1.630 ± 0.035
Magnesium (Mg)	1.710 ± 0.015	1.630 ± 0.059
Manganese (Mn)	0.303 ± 0.007	0.303 ± 0.012
Sodium (Na)	0.310 ± 0.010	0.347 ± 0.015
Zinc (Zn)	0.227 ± 0.003	0.217 ± 0.003
*Non-metallic minerals*		
Phosphorus (P)	0.777 ± 0.015	0.720 ± 0.015
Sulfur (S)	0.253 ± 0.003	0.267 ± 0.009

The values are expressed as the mean ± SEM. Analyses were performed in triplicate.

**Table 2:** In vitro antioxidant activity of the ethanolic extract of cerumen from *Geotrigona* sp. (EEC-G) and from *Tetragonisca fiebrigi* (EEC-T).

Sample	DPPH^•^	ABTS^•+^
	IC_50_ (mg/mL)	IC_50_ (mg/mL)
Ascorbic acid	0.004 ± 0.00029	0.003 ± 0.00006
BHT	0.031 ± 0.005	0.009 ± 0.0009
EEC-G	1.001 ± 0.062	0.496 ± 0.040
EEC-T	1.251 ± 0.068	0.254 ± 0.023

BHT: butylated hydroxytoluene; DPPH^•^: 2,2-diphenyl-1-picrylhydrazyl; ABTS^•+^: 2,2′-azino-bis(3-ethylbenzothiazoline-6-sulfonic acid); IC_50_: concentration capable of inhibiting 50% of the free radical. The values are expressed as the mean ± SEM. Three independent experiments were performed in duplicate.

## Data Availability

The data presented in this study are available on request from the corresponding author.
